# Severe Type B Lactic Acidosis in a Rare and Aggressive HIV-Related Lymphoma

**DOI:** 10.1155/2019/4642925

**Published:** 2019-08-20

**Authors:** John Harwood Scott, Ashish P. S. Bains, Timothy D. Lindsay, Xiaofeng Zhao, Michael E. Bromberg

**Affiliations:** ^1^Department of Medicine, Section of Internal Medicine, Lewis Katz School of Medicine at Temple University Hospital, Philadelphia, PA 19140, USA; ^2^Department of Pathology and Laboratory Medicine, Lewis Katz School of Medicine at Temple University Hospital, Philadelphia, PA 19140, USA; ^3^Department of Medicine, Section of Hematology, Lewis Katz School of Medicine at Temple University Hospital, Philadelphia, PA 19140, USA

## Abstract

We describe the prognostic implication and aggressive clinical course of lymphoma-related lactic acidosis in a rare HIV-related lymphoma. Patient was diagnosed with plasmablastic lymphoma and developed severe lactic acidosis, and was treated on the medical floor and in the medical intensive care unit. Her lactic acidosis was considered to be type B, secondary to her underlying lymphoma since she never had an infectious source, hypovolemic state, or low/high cardiac-output state. The mechanism of the lymphoma-related lactic acidosis is from altered cellular metabolism, thought to aid in lymphoma proliferation, rather than tissue hypoperfusion. It is a rare complication of aggressive lymphomas and signifies a poor prognosis. Patients having this complication should be considered for close monitoring and management in an intensive care unit until definitive treatment (i.e., chemotherapy) can be implemented.

## 1. Introduction

Lactic acidosis is a rare feature of malignancy and carries a poor prognosis with a high mortality rate. The underlying mechanism for lactic acidosis in aggressive lymphomas might differ and could be secondary to “the Warburg effect” and classifiable as type B. Management of Type B lactic acidosis centers on the treatment of the underlying etiology and hinges on prompt treatment of the underlying malignancy. Plasmablastic lymphoma is an HIV-related malignancy, accounting for approximately 2% of all HIV-related lymphomas, although the true incidence is unknown and this lymphoma can also occur in HIV-negative patients who are immunosuppressed or thought to be immunosenescence. We discuss the etiology and management of a rare occurrence of both plasmablastic lymphoma and associated type B lactic acidosis.

## 2. Case

The patient is a 29-year-old female with a history of HIV on highly active antiretroviral therapy (HAART) who presented to the hospital with complaints of nausea, vomiting, and diarrhea. Five days prior to admission, the patient developed back pain, which she described as cramping, constant, and radiating throughout her back despite NSAID use. Two days prior to admission, she began to experience fatigue and subjective fevers and then developed nausea with nonbloody/nonbilious vomiting and nonbloody diarrhea that prompted her to present to the Emergency Department.

The patient reported compliance with her HAART and denied recent changes to her regimen. On review of systems, she reported an approximately 45 lbs. unintentional weight loss over 9 months. She recently had two prolonged hospitalizations for pneumonia with *Streptococcus pneumoniae* bacteremia and perianal abscess that required incision and drainage.

Admission vitals were significant for tachycardia. Her physical exam was notable for diffuse abdominal tenderness with hepatosplenomegaly and diffuse lymphadenopathy that was most appreciable in the submandibular, supraclavicular, and inguinal regions. Her laboratory studies were remarkable for a bicarbonate of 16 mmol/L (reference lab values 22–32 mmol/L), anion gap of 21 mmol/L (reference lab value 6–16 mmol/L), and lactate of 7.3 mmol/L (reference lab value 0.5–2.2 mmol/L). A complete blood count showed a hemoglobin of 10 g/dL (reference lab values 14–17.5 g/dL), platelet count of 87 K/mm^3^ (reference lab values 150–450 K/mm^3^), and white cell count of 8 K/mm^3^ (reference lab values 4–11 K/mm^3^). Her arterial pH was 7.26 (reference lab values 7.35–7.46).

Initial imaging, which included a computed tomography (CT) scan of the chest, abdomen, and pelvis, revealed extensive lymphadenopathy above and below the diaphragm along with pleural-based masses, marked hepatosplenomegaly, and a large retroperitoneal mass (4.6 × 9.2 × 9.1 cm) encasing the abdominal aorta and inferior vena cava. There was also significant progression of her lymphadenopathy when compared with a CT scan from a month prior to admission, which showed only mild pelvic lymphadenopathy thought secondary to a perianal abscess.

On admission, the patient was aggressively volume resuscitated, pan-cultured, and placed on broad spectrum antibiotics. However, her lactate level increased to 11.5 mmol/L and she became febrile. Blood, urine, and sputum cultures were unrevealing. The patient was seen by the surgery and infectious disease services who advised against any surgical procedure or continuing antibiotics, respectively.

**Figure 1:**
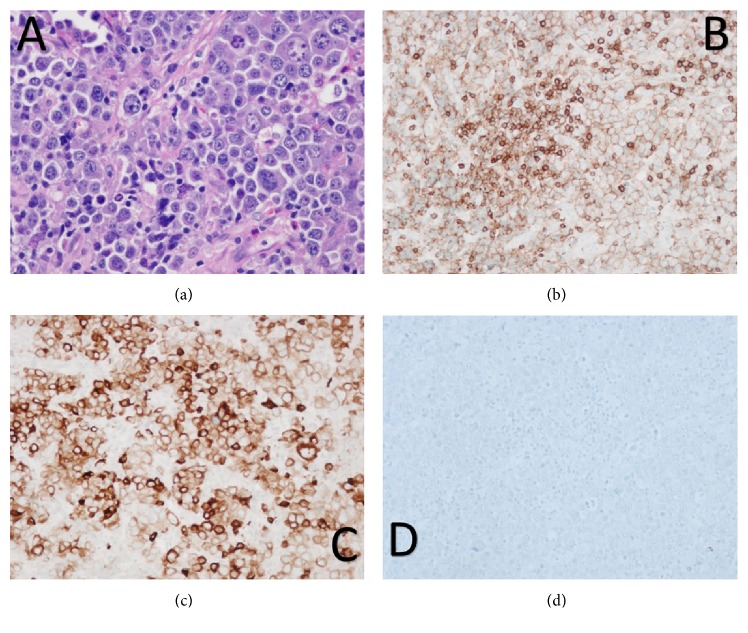
Plasmablastic lymphoma: (a) large pleomorphic neoplastic cells with prominent nucleoli and abundant cytoplasm, H&E stain (40x); (b) weak positive CD45 immunostain (20x); (c) positive CD79a immunostain (20x); (d) negative CD20 immunostain (20x).

On hospital Day #2 (HD2), the patient remained hemodynamically stable with only fevers and tachycardia that was refractory to volume resuscitation. She underwent an ultrasound-guided fine needle aspirate of a left supraclavicular lymph node with flow cytometry, which was nondiagnostic. She subsequently underwent an excisional biopsy of left cervical lymph node. The lymph node pathology showed sheets of large, highly pleomorphic neoplastic cells with abundant cytoplasm, irregular nuclei, and variably prominent nucleoli (Figure 1). Ki67 proliferation rate was found to be approximately 85%. Immunohistochemical stains showed a plasmacytic phenotype with positive expression of CD79A, CD45 (weak), CD30, MUM1, and CD138 while negative staining for CD20 and PAX5 (Figure 1). Other negative stains included CD5, BCL2, BCL6, cyclinD1, and ALK1. In situ hybridization studies showed weak lambda light chain restricted expression and the neoplastic cells were positive for Epstein-Barr virus-encoded small RNA (EBER). The pathology findings were diagnostic of plasmablastic lymphoma (PBL). Bone marrow biopsy demonstrated lymphoma involvement with scattered CD138 positive large neoplastic cells which were also positive for EBER by in situ hybridization.

**Figure 2:**
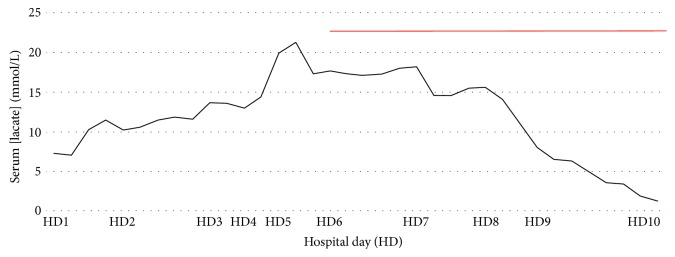
Lactic acid levels (in mmol/L) of patient starting at admission until resolution. Red bar indicates administration of EPOCH chemotherapy that finished on hospital day 11.

The patient's lactate continued to increase from 7.3 mmol/L on admission to a peak of 21.3 mmol/L on hospital HD5 (Figure 2). Her lactic acidosis was considered to be secondary to her underlying lymphoma since she never had an infectious source, hypovolemic state, or low/high cardiac-output state. In addition, her HAART was held for the possibility of medication-related acidosis. Following her lymph node biopsy, the patient was transferred to the intensive care unit (ICU) where she was kept on a continuous bicarbonate-infusion to maintain pH above 7.2 and closely monitored for possible renal-replacement therapy. The lactic acidosis did not resolve, however, until after the she was initiated on chemotherapy on HD6, which consisted of etoposide, prednisone, vincristine, cyclophosphamide, and doxorubicin (EPOCH) as seen in Figure 2. Following the administration of EPOCH, the patient was transferred out of the ICU. She tolerated the chemotherapy well, and subsequently was discharged in a stable condition.

The patient underwent two more cycles of EPOCH. Cycle #2 was complicated by multifocal pneumonia. She was then lost to follow-up for three weeks before returning for Cycle #3. During Cycle #3, she experienced persistent neutropenic fevers and severe thrombocytopenia. In addition, she required transfer to the ICU for altered mental status requiring intubation and hypotension requiring vasopressors. The etiologies for both were not fully known. She had been having occipital headaches with blurred vision and agitation that required opioid analgesia and benzodiazepam sedation. In addition, she was having high fevers, tumor lysis syndromes, shock liver, and return of a lactic acidosis. A CNS work-up included brain MRI and lumbar puncture which were negative for evidence of infection or lymphomatous CNS involvement. No other infectious source was established. Additional CT imaging revealed disease progression with worsening thoracic and abdominal lymphadenopathy. She was eventually transferred out of the ICU after slow improvement of her mental status with empiric broad spectrum antibiotics and removal of sedating medications.

The patient, however, developed worsening pancytopenia and hypoxic respiratory failure in addition to persistent fevers, elevated lactate-dehydrogenase levels, and progressively elevated lactic acid levels. A bone marrow biopsy was performed and confirmed persistent PBL involvement. In addition, a follow-up CT scan of the chest demonstrated an ill-defined left hilar mass (that was occluding and collapsing the lingual airways and segment) along with worsening diffuse lymphadenopathy and diffuse lymphedema in the lungs.

Salvage chemotherapy which consisted of cyclophosphamide, bortezomib, dexamethasone (CyBorD) was initiated. However, the patient developed multifocal, hospital-acquired pneumonia and had worsening lymphadenopathy and tonsillar swelling despite treatment. Further salvage therapy with dexamethasone, bortezomib, and daratumumab was administered. Daratumumab was chosen because of its efficacy in relapsed/refractory multiple myeloma and lack of myelosuppression [1]. However, she again required transfer to the ICU for hypercapnic and hypoxemic respiratory failure, became progressively somnolent, and required intubation. The patient then became markedly hypotensive despite multiple transfusions of blood products and aggressive volume resuscitation as well as vasopressor support. She ultimately went into ventricular fibrillation arrest and expired. Her death was 137 days after her initial diagnosis of her lymphoma.

## 3. Discussion

Lactic acidosis is a rare feature of malignancy, the majority of which are lymphomas, and conveys a poor prognosis [2]. Literature reviews of case reports and case series suggest a high mortality rate of 76–81% [2, 3]. Not surprisingly, survival and resolution of the lactic acidosis hinge on prompt treatment of the underlying malignancy [3].

Lactic acidosis is typically the result of tissue hypoperfusion resulting from a variety of serious clinical conditions such as shock, sepsis, heart failure, end-organ ischemia, or hypovolemia. This etiology grouping is classified as Type A lactic acidosis. Lactic acidosis in the absence of tissue hypoperfusion is classified as Type B. Given the mortality associated with Type A lactic acidosis, any discovered lactic acidosis should be presumed Type A until otherwise ruled out with clinical and laboratory findings [4–7].

Type B lactic acidosis in lymphoma is secondary to “the Warburg effect” [4–10]. Warburg observed that malignant cells utilized glycolysis over oxidative phosphorylation for energy production, regardless of how oxygen rich the environment [4, 5, 8, 10]. This process is referred to as aerobic glycolysis and, like anaerobic glycolysis, pyruvate is not broken down into acetyl CoA for the Krebs cycle, but instead is converted to lactic acid for generation of NADH. The efficiency of aerobic glycolysis in ATP and NADPH generation is far inferior to oxidative phosphorylation, yet this process appears to convey some cellular growth advantage since even single cell organisms utilize it prior to cell replication. While the mechanism is not fully known, aerobic glycolysis is thought to aid in biosynthesis needed for cell proliferation [4, 5, 8, 11].

The accumulation of lactic acid is due not only to a high burden of lymphoma cells undergoing aerobic glycolysis, but also from impaired hepatic clearance of lactate, which is thought to be down regulated from other malignancy-related factors. Depletion of thiamine, an important cofactor for pyruvate to enter the Kreb's cycle, has been suspected for further shunting of glucose substrates to lactate and its repletion has sometimes helped correct the acidosis [2, 8]. Consumption of glucose for lymphoma cell replication resulting in lactic acidosis is sometimes associated with persistent hypoglycemia. There are numerous case reports of patients requiring aggressive intravenous repletion of glucose along with management of their lactic acidosis [2, 5, 7–9].

Management of Type B lactic acidosis centers on the treatment of the underlying etiology [4, 5, 8, 12–17]. Chemotherapy is typically the treatment for lymphoma-related Type B lactic acidosis [4, 5, 8, 12–16]. Tissue diagnosis and pathology, however, is required prior to initiation of chemotherapy and temporizing measures are needed for the initial management of the acidosis. Continuous bicarbonate infusions are a key therapeutic modality but require monitoring for volume overload and hypernatremia. Alternatively, continuous-veno-venous-hemodialysis and sustained low efficiency dialysis (SLED) have been utilized to remove lactate and replace bicarbonate [18]. There is evidence to suggest SLED is superior to bicarbonate infusion as the former was not associated with volume overload, hypernatremia, or a paradoxical increasing in lactic acid production [18]. Thiamine repletion has also become part of the empiric treatment based on its role pyruvate metabolism [2, 14, 19].

PBL is an HIV-related malignancy, accounting for approximately 2% of all HIV-related lymphomas, although the true incidence is unknown [20]. This lymphoma also occurs in HIV-negative patients who are immunocompromised as a result of immunosuppression in solid organ transplant, autoimmune disease, and in elderly patients with presumptive immunosenescence. The diagnosis of PBL is often challenging as it shares pathologic features of plasmablastic myeloma or lymphomas with plasmablastic morphology. The malignant cells are commonly found to be infected with EBV, which is likely a crucial factor in the lymphoma's pathogenesis along with *MYC* translocation [20–24]. PBL is an aggressive lymphoma with a high incidence of extra-nodal involvement at presentation. In HIV-positive patients, median overall-survival (OS) for PBL is poor with treated patients having 15 month OS compared with 3 month OS in those not receiving treatment [20].

Treatment of PBL has stemmed from knowledge in treating aggressive HIV-related lymphomas, and currently it is recommended to use more intensive chemotherapy regimens than CHOP (cyclophosphamide, doxorubicin, vincristine, prednisone) [25, 26]. Chemotherapy regimens for PBL include dose-adjusted EPOCH, HyperCVAD (cyclophosphamide, doxorubicin, vincristine, prednisone), and modified CODOX-M/IVAC (cyclophosphamide, vincristine, doxorubicin, high-dose methotrexate/ifosfamide, etoposide, and high-dose cytarabine) [25, 26]. None of these regimens have been shown to be superior. However, EPOCH is thought to be more efficacious than CHOP in some HIV-related lymphomas [27]. Given PBL's aggressiveness, CNS prophylaxis with methotrexate or cytarabine has been recommended. Autologous hematopoietic stem cell transplantation with high-dose chemotherapy has been shown to improve OS when implemented following first remission [27].

Despite the rare occurrence of both PBL and lactic acidosis with lymphoma, there are two other case reports of lactic acidosis in PBL [21, 23]. However, it is not fully known how prevalent the association of lactic acidosis is with PBL.

## 4. Conclusion

Severe lactic acidosis is a rare complication of aggressive lymphomas and signifies a poor prognosis. Patients having this complication should be considered for close monitoring and management in an intensive care unit until definitive treatment (i.e., chemotherapy) can be implemented. The mechanism of the lymphoma-related lactic acidosis is from altered cellular metabolism, thought to aid in lymphoma proliferation, rather than tissue hypoperfusion.
